# Supplementary material for: Co‐activation of Selective Nicotinic Acetylcholine Receptor Subtypes Is Required for Neuroprotection against Alzheimer's Disease
Source: Adv Sci (Weinh). 2026 Sep 27:e77858. Online ahead of print. doi: 10.1002/advs.77858 (PMC13616367; doi:10.1002/advs.77858)
Supplement: Supplementary file 1 [file ADVS-9999-e77858-s002.pdf]

Supplementary Fig 1.

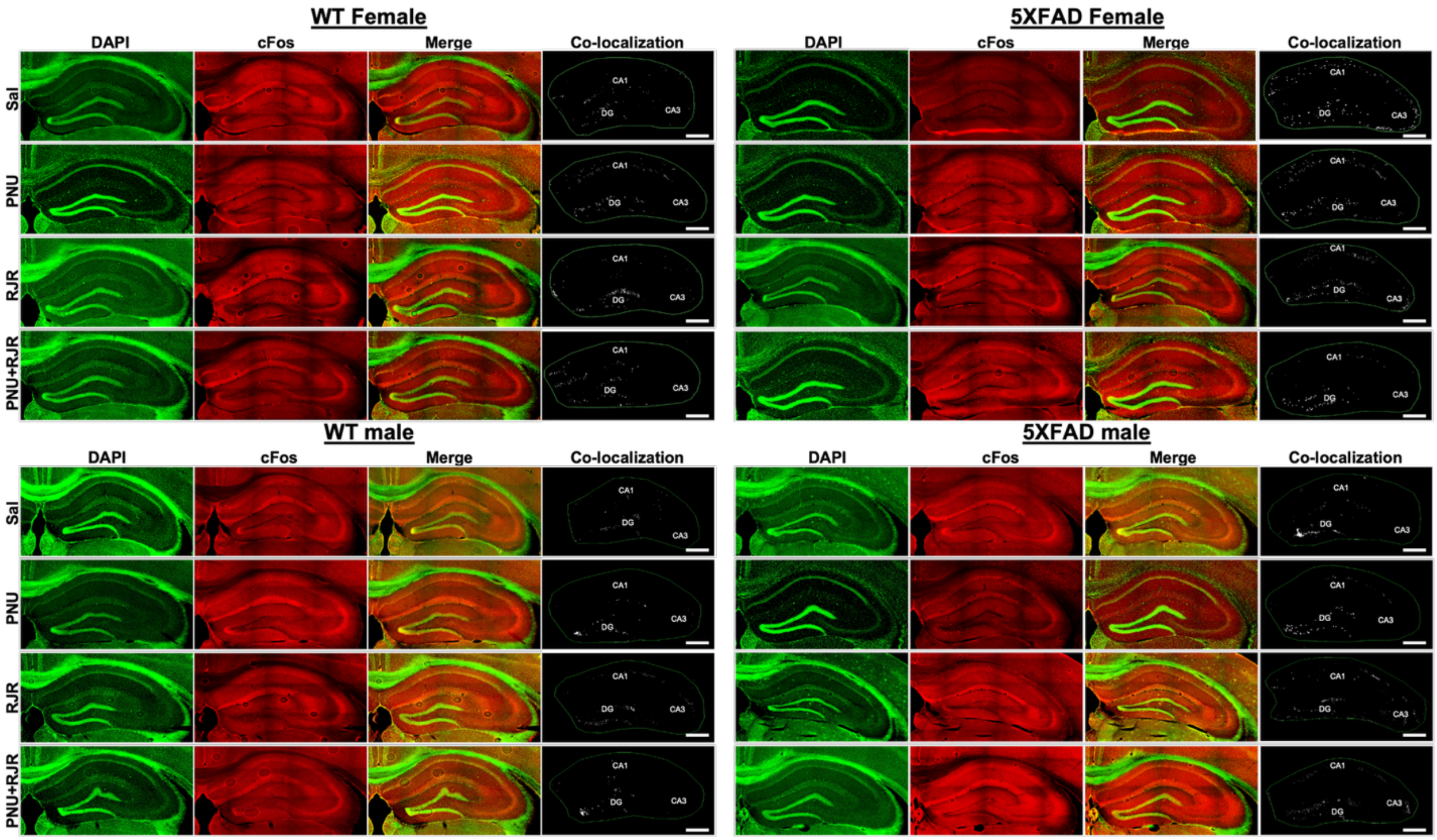

Representative hippocampal sections from the indicated experimental groups in Fig. 3b showing DAPI staining (green; left), c-Fos immunoreactivity (red; middle-left), and merged images (middle-right). Co-localization of c-Fos immunoreactivity with DAPI-labeled nuclei (c-Fos+/DAPI+ cells) identifies c-Fos-positive activated cells, including pyramidal neurons within the hippocampus. Corresponding hippocampal maps (right) show the distribution of c-Fos+/DAPI+ cells within the CA1, dentate gyrus (DG), and CA3 subregions. Outlines show hippocampal region of interest (ROI). Scale bars indicate 500 μm.

Supplementary Fig 2.

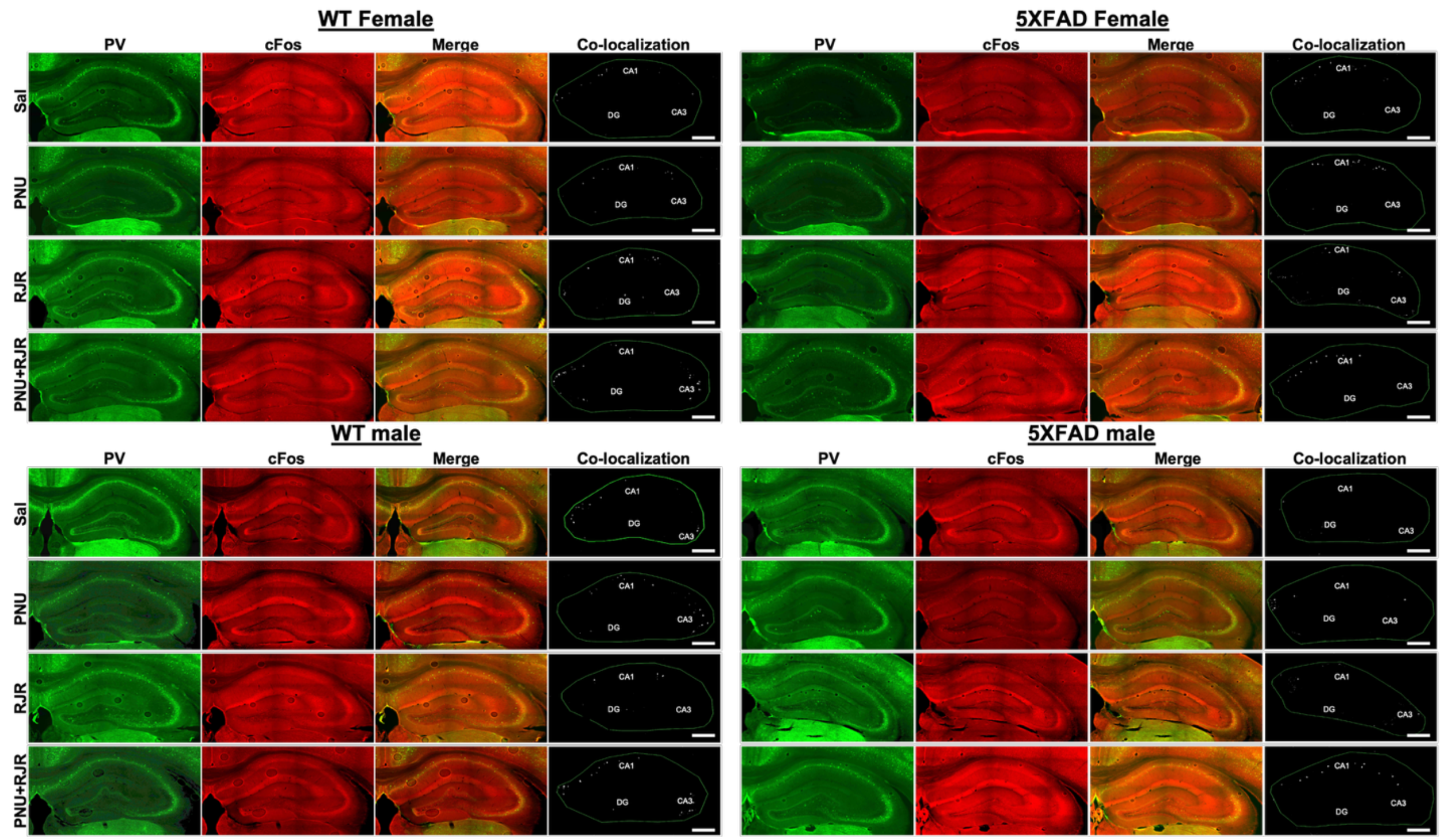

Representative hippocampal sections from the indicated experimental groups in Fig. 3c showing PV staining (green; left), c-Fos immunoreactivity (red; middle-left), and merged images (middle-right). Co-localization of c-Fos immunoreactivity with PV+ interneurons (c-Fos+/PV+ cells) identifies c-Fos-positive activated PV+ cells within the hippocampus. Corresponding hippocampal maps (right) show the distribution of c-Fos+/PV+ cells within the CA1, dentate gyrus (DG), and CA3 subregions. Outlines show hippocampal region of interest (ROI). Scale bars indicate 500 μm.

Supplementary Fig 3.

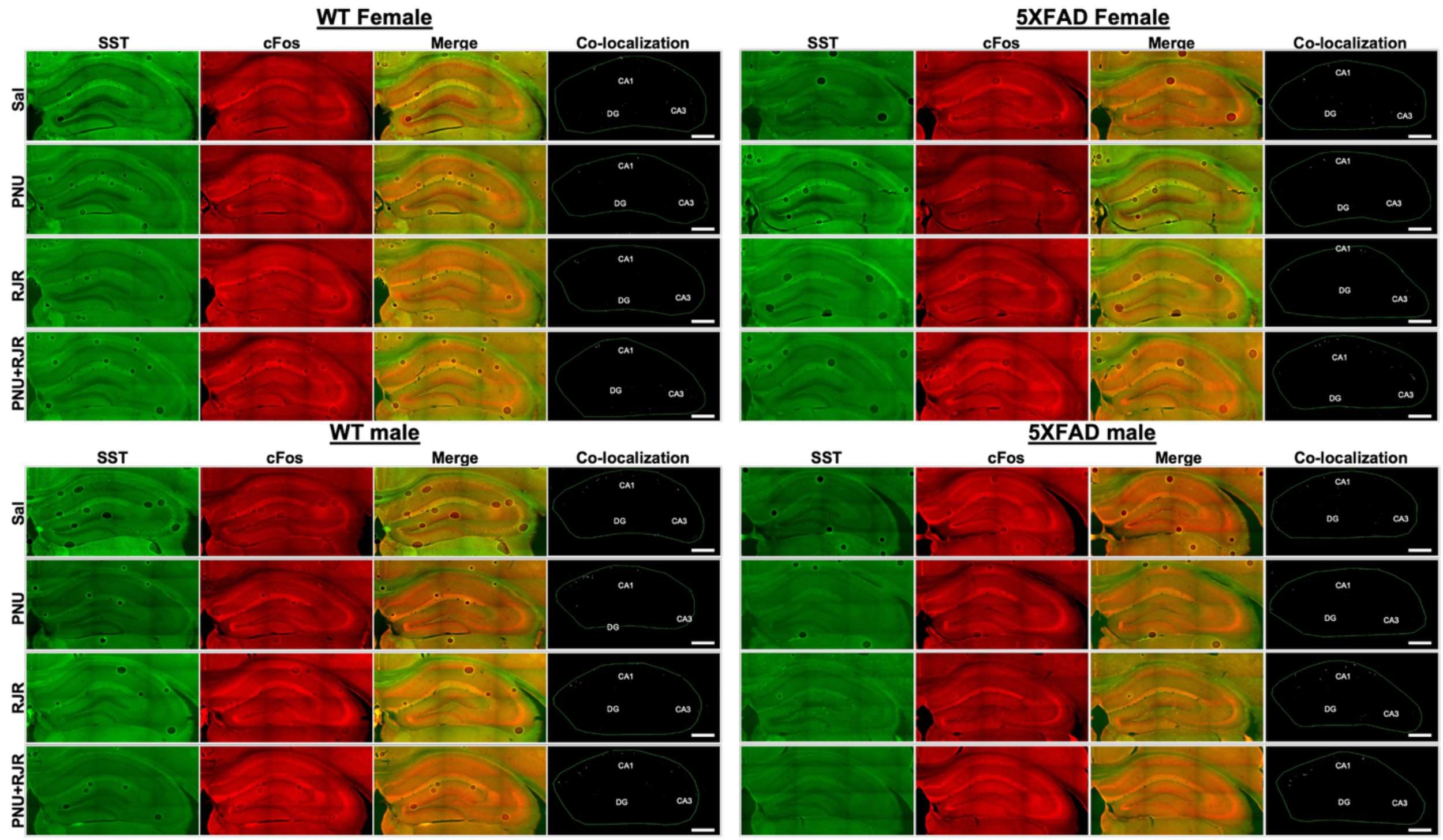

Representative hippocampal sections from the indicated experimental groups in Fig. 3d showing SST staining (green; left), c-Fos immunoreactivity (red; middle-left), and merged images (middle-right). Co-localization of c-Fos immunoreactivity with SST+ interneurons (c-Fos+/SST+ cells) identifies c-Fos-positive activated SST+ cells within the hippocampus. Corresponding hippocampal maps (right) show the distribution of c-Fos+/SST+ cells within the CA1, dentate gyrus (DG), and CA3 subregions. Outlines show hippocampal region of interest (ROI). Scale bars indicate 500 μm.
